# Cluster-randomised trial on participatory community-based outdoor physical activity promotion programs in adults aged 65–75 years in Germany: protocol of the OUTDOOR ACTIVE intervention trial

**DOI:** 10.1186/s12889-018-6124-z

**Published:** 2018-10-23

**Authors:** Karin Bammann, Carina Drell, Lena Lotte Lübs, Imke Stalling

**Affiliations:** Institute for Public Health and Nursing Sciences (IPP), University of Bremen, Bremen, Germany

**Keywords:** Physical activity, Older adults, Health promotion, Community-based intervention, PRECEDE-PROCEED model

## Abstract

**Background:**

Despite its well-known benefits for health and well-being physical activity levels are insufficient and declining with age in Germany. Physical activity promotion programs for older adults are often not successful, one reason is insufficient relevance of intervention measures for the study population. Community-based participatory research (CBPR) is a possible key strategy for tailoring more meaningful intervention programs to a specific community. However, evidence for the effectiveness of CBPR in the general population is scarce. This study aims to formally evaluate the efficacy of a CBPR approach for developing and implementing an outdoor physical activity program for older adults.

**Methods/design:**

The OUTDOOR ACTIVE intervention trial is a cluster-randomised intervention study carried out in a random sample of eight subdistricts in the city of Bremen, Germany. The eight subdistricts are grouped into four homogenous pairs with regard to socioeconomic level and land use mix of the subdistrict. Within the pairs, the subdistricts are assigned randomly to the two study arms: participatory development and implementation of a community-based program to promote outdoor physical activity (intervention) versus no intervention (controls). For evaluation, a survey is carried out before (baseline) and after (follow-up) the intervention period. The measurements include 7-day accelerometer measurement, physical fitness test, blood pressure, basic anthropometry, and self-administered questionnaire.

**Discussion:**

The OUTDOOR ACTIVE intervention trial will provide detailed information on PA intervention for older adults in an urban setting. Through the participatory nature of the study it will provide valuable insights into drivers and barriers to PA in this group, and it will inform policy makers and other stakeholders how to benefit from the results.

**Trial registration:**

German Clinical Trials Register DRKS00015117 (Date of registration 17-07-2018).

## Background

The current demographic change in Europe is characterized by decreasing birth rates, rising life expectancy and an ageing population. This development is posing challenges on private, social and societal levels, and healthy ageing has become one key strategy to fight the expected added burden of the health system [1]. Physical activity (PA) is declining with age, and it is less prevalent in women compared to men and in lower compared to higher socioeconomic groups [2]. Despite its known benefits for health, more than two-thirds of the age group 65 years and older do not meet minimal PA recommendations in Germany [3] and raising these levels, preferably in community-based or neighborhood-based settings, forms one of the seven national specific health targets in the area of healthy ageing [4]. Cochrane reviews on PA promotion have repeatedly criticized the lack of intervention studies with sound methodology using objective PA measurements for evaluation [5–7].

Various factors of all ecological levels (intrapersonal, interpersonal, and environmental) contribute to the uptake and maintenance of any type of PA [8]. However, due to a lack of longitudinal studies and studies using objective PA measurements, a differentiation between correlates and determinants is barely possible [7, 8]. Factors that could play a causal role for PA in adults in general are perceived fitness, intention to exercise, self-efficacy, social support, PA history, and other psychosocial variables [8–11]. Apart from these intra- and interpersonal characteristics, the engagement in PA is strongly dependent on environmental factors [8, 12–14] and environmental intervention has been shown to be potentially successful previously [15]. Research in older adults is still scarce; a review from 2004 implies that among environmental factors, safety and aesthetics could play the most important roles in older age groups [16]. A review by van Cauwenberg and colleagues on determinants for PA [17] found inconsistent results for the age group 65+, which the authors attribute to “methodological issues within this developing research field”. Only two of the 29 included studies were using objective PA measurements, and most studies failed to differentiate between types of activity. The latter would be important for detecting associations between PA and environmental factors [17]. As any engagement in PA, outdoor PA has direct beneficial health effects on muscle strength, motor skills and cardiorespiratory fitness. Outdoor PA also has indirect effects that are not attributed to the physical movement alone, including higher Vitamin D levels, mental wellbeing and raised emotional scores through exposure to sensory engagement [18]. EEC readings show the direct impact of the PA environment [19]. In a recent study, older adults who were physically active outdoors at least once a week showed higher levels of PA compared with those who were physically active indoors only [20]. Moreover, outdoor PA gives the opportunity for social interaction and does not require sports facilities.

The success of PA promotion programs is heterogeneous [21, 22] and depends amongst others on the type of intervention and presence of a methodological framework for the development of the intervention [23]. Community-based participatory research (CBPR) frameworks show great potential for PA intervention as they involve the community, especially if they follow an ecological model [10]. CBPR have been shown to be effective for work-related health promotion [24], and they are useful to reduce health disparities [25]. Experience with CBPR in the development of PA promotion programs in older adults is limited. Despite the poor evidence base, PA interventions following a social ecological approach and integrating individual and environmental levels are considered to be most effective [8–11].

The OUTDOOR ACTIVE study is part of the *Physical Activity And Health Equity: Primary Prevention For Healthy Ageing (AEQUIPA)* project, a regional prevention research network in Northwest Germany funded by the German Federal Ministry of Education and Research (BMBF). The AEQUIPA research network includes several interlinked projects which employ theory-based empirical research methods to develop, implement and evaluate PA and mobility interventions for older adults aged 65–75 years. The network’s overall aim is to strengthen the evidence base for PA in the context of healthy ageing [26]. AEQUIPA is in its second funding phase (02/2018–01/2021), the first funding phase started in 02/2015. In the OUTDOOR ACTIVE pilot study, which took place during the first funding phase, a community-based physical activity program was developed in one urban district in the city of Bremen, Germany, where a CBPR approach, the PRECEDE-PROCEED model (PPM), was used [27]. Based on the experiences, a short track PPM for participatory development of PA programs in older adults in urban settings was developed. Moreover, an open toolbox of ready-to-use intervention components to be used in the participatory process was started and will be continuously updated.

The objective of the OUTDOOR ACTIVE intervention trial is to formally test and investigate efficacy of the developed short track PPM. For this, a cluster randomized trial (CRT) will be carried out in eight random subdistricts in the city of Bremen, Germany. In four of the randomly selected subdistricts, the short track PPM will be applied and the resulting intervention program implemented, the other four randomly selected subdistricts will serve as controls, where no intervention is taking place.

## Methods/design

### Study setting and participants

**Fig. 1:**
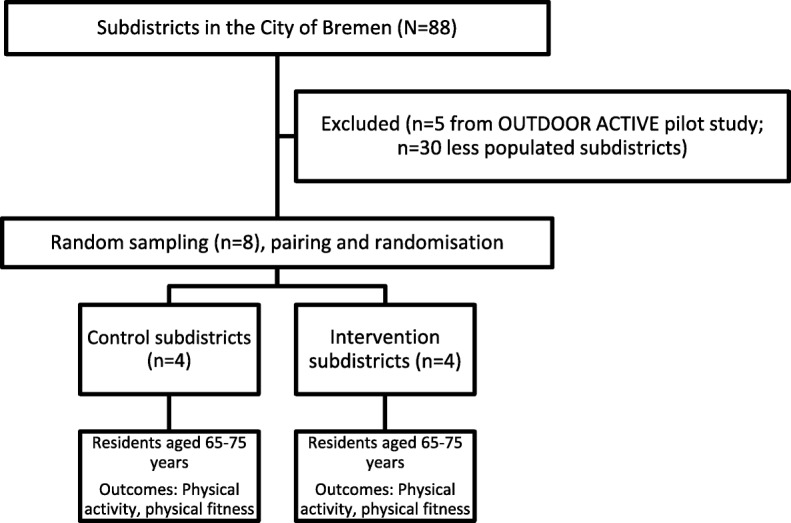
The OUTDOOR ACTIVE intervention trial

The OUTDOOR ACTIVE intervention trial will be taking place in randomly selected subdistricts of the city of Bremen. The city municipality of Bremen is located in north western Germany with around 560,000 inhabitants. Bremen is organised hierarchically into 5 boroughs, 23 urban districts and 88 subdistricts. The first randomization unit are the subdistricts, where less populated subdistricts (with less than 500 inhabitants aged 65–75 years) and the five subdistricts of the OUTDOOR ACTIVE pilot study are excluded, leaving 53 subdistricts eligible for the study (see Fig. 1). The subdistricts of the city of Bremen are highly heterogeneous e.g. with respect to SES indicators (e.g. proportion of residents with low school education ranging from 18.5 to 89.2%, medium taxable income ranging from 10,069 € to 35,995 €), life expectancy (ranging from 72.4 [78.2] years to 81.0 [85.3] years in males [females]) or land use mix (e.g. proportion of recreational area ranging from 0.1 to 66.9%; all data from [28]).

Persons eligible for the study are non-institutionalized adults, aged 65–75 years residing in the sampled subdistricts. Depending of the number, either a simple random sample or the full sample of eligible participants will be invited to the study. Persons not able to give consent will be excluded.

### Interventions

The eight subdistricts are manually grouped into homogenous pairs with regard to community socioeconomic status and land mix use. From these pairs, one intervention subdistrict is randomly chosen, the remainder serves as control subdistrict.

A physical activity program tailored to the specific situation in the respective subdistrict will be developed in each of the intervention subdistricts with active involvement of the general public and other stakeholders. For tailoring, data from situational analysis, baseline surveys, and participatory actions (community forum, workshops, and excursions) is used. Each development step is communicated and discussed in the community and tested for its feasibility. The intervention material is being developed as part of an ecological model [16]. Implementation will be done with the help of local stakeholders and key actors of the population to ensure sustainability. A community round table with all stakeholders will take place throughout the intervention phase in the intervention subdistricts.

### Outcomes

For the formal CRT evaluation, baseline and follow up surveys are carried out in the eight study subdistricts. These include 7-days measurements of physical activity (3D accelerometer, ActiGraph, Pensacola (FL)) and fitness (modified Senior Fitness Test [29]) a short physical examination (blood pressure, short anthropometry) and a self-administered questionnaire on intrapersonal, interpersonal, and environmental determinants of physical activity. Training sessions on fitness test and physical examination are held regularly for the field staff to ensure standardised measurements.

The primary outcome is amount of PA measured by accelerometer in average counts per minutes (CPM). The secondary outcome is physical fitness. The tests are handgrip strength, chair stand, 2-min step, back scratch, sit and reach, and flamingo balance test. Further secondary outcomes are time spent outdoors in minutes per day (measured by questionnaire) and inactivity in hours per day (measured by questionnaire). The impact evaluation will include identified key determinants. Evaluation of efficacy and possible adverse effects of the PA promotion will be done stratified by sex. Mixed models will be used to account for the clustered structure of the data introduced by the two-stage sampling design.

### Participant timeline, blinding and sample size

**Fig. 2:**
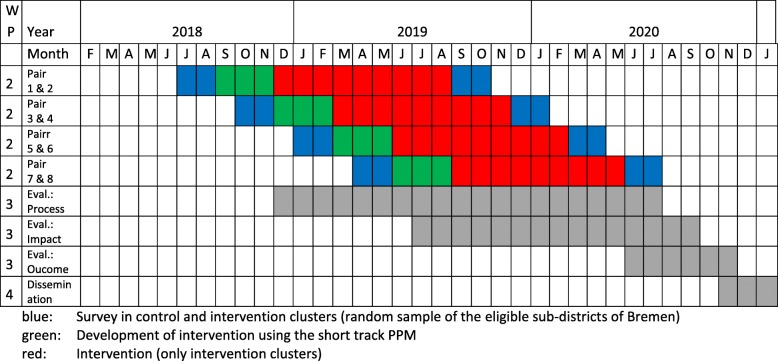
Timeline of the OUTDOOR ACTIVE intervention trial

Participant timeline is depicted in Fig. 2. The four subdistrict pairs will be included consecutively and undergo identical procedures and time schedules. This design will help to control for seasonal or weather effects. Each pair starts and ends with baseline and follow up surveys in the subdistrict. In the intervention subdistrict, development and implementation of the intervention will take place. The address data will be obtained by the registry office of the city of Bremen. Prospective participants will be recruited via written and telephone contact. A detailed written feedback will be sent to all participants after the follow up survey.

The sample size calculation is based on data gathered during the OUTDOOR ACTIVE pilot study. Accelerometer-based average counts per minute (CPM) will be used for evaluation. Mean CPM ranged from 1587.5 (SD 470.7) to 1697.0 (SD 440.5) in the five highly heterogeneous subdistricts (ICC = 0.0024). CPM were consistently higher in women than in men with only moderate differences for SES. In the OUTDOOR ACTIVE intervention trial, a mean difference of 150 CPM (equivalent to standardized effect size of 0.33) is targeted. Assuming a fixed number of clusters (four intervention, four control), 204 participants will be needed in each study arm (significance level 5% two-sided, power 90%) summing up to 808 for sex-stratified trial evaluation. Assuming a fixed proportion of 30% for dropouts, 144 participants (72 female, 72 male) are needed in each of the subdistricts at baseline.

Since no intervention is developed in the control subdistricts, and active involvement of the study participants is required in the intervention subdistricts, blinding was not a feasible option for the intervention trial. Instead, for communication with the public, the study is separated into two parts: one part containing the surveys (“BUTEN AKTIV Gesundheitsuntersuchung” OUTDOOR ACTIVE health surveys), which take place in all eight subdistricts and focuses on longitudinal aspects of the ageing process; the second part containing the intervention development and implementation, takes only place in the four intervention subdistricts (“BUTEN AKTIV vor Ort” OUTDOOR ACTIVE on-site). Thus, participants of the survey might or might not be aware of the intervention development.

## Discussion

PA is an important component for healthy ageing with many documented benefits both for society and the individual [30]. Thus, the proportion of persons meeting the recommendations for PA should be as high as possible in all age groups. In older adults, PA promotion is especially important, since albeit the potential gain for this group is large, engagement in PA is decreasing with age [31]. Especially outdoor PA, with its added external stimuli is a valuable health resource in older age [32].

In the OUTDOOR ACTIVE intervention trial, we formally evaluate the efficacy of a participatory community-based approach for tailoring an outdoor PA promotion for older adults to a random subdistrict with its given actors and structures. If successful, partners will be sought to implement the intervention in all subdistricts of Bremen such that control subdistricts will also directly benefit from the trial. Moreover, the approach and a ready-to-use toolbox for applying it will be published and made available to other communities. The OUTDOOR ACTIVE intervention trial will provide detailed information on PA intervention for older adults in an urban setting. The use of an objective method for the assessment of the main outcome, physical activity, helps eliminating recall and social desirability bias [33]; and ensures international comparability of the results. Through the participatory nature of the study it will provide valuable insights into drivers and barriers to PA in this group, and it will inform policy makers and other stakeholders how to benefit from the results.

## Abbreviations

AEQUIPA: Physical activity and health equity: primary prevention for healthy ageing
BMBF: Federal ministry of education and research
CBPR: Community-based participatory research
CPM: Counts per minute
CRT: Cluster randomized trial
ICC: Intraclass correlation coefficient
PA: Physical activity
PPM: PRECEDE-PROCEED model
SD: Standard deviation
SES: Socio-economic status
